# NF-κB Regulation by Gut Microbiota Decides Homeostasis or Disease Outcome During Ageing

**DOI:** 10.3389/fcell.2022.874940

**Published:** 2022-07-01

**Authors:** Shuning Zhang, Soumyajeet Paul, Parag Kundu

**Affiliations:** ^1^ Laboratory for Microbiota-Host Interactions, The Center for Microbes, Development and Health, Institut Pasteur of Shanghai, Chinese Academy of Sciences, Shanghai, China; ^2^ University of Chinese Academy of Sciences, Beijing, China

**Keywords:** gut microbiota, dysbiosis, NF-κB, ageing, inflammaging, homeostasis

## Abstract

Human beings and their indigenous microbial communities have coexisted for centuries, which led to the development of co-evolutionary mechanisms of communication and cooperation. Such communication machineries are governed by sophisticated multi-step feedback loops, which typically begin with the recognition of microbes by pattern recognition receptors (PRRs), followed by a host transcriptional response leading to the release of effector molecules. Our gastrointestinal tract being the main platform for this interaction, a variety of host intestinal cells tightly regulate these loops to establish tolerance towards the microbial communities of the gut and maintain homeostasis. The transcription factor, nuclear factor kappa B (NF-κB) is an integral component of such a communication apparatus, which plays a critical role in determining the state of homeostasis or inflammation associated with dysbiosis in the host. Here we outline the crucial role of NF-κB in host response to microbial cues in the context of ageing and associated diseases.

## Introduction

The recent realization that microbiota, which colonizes multiple niches of our body is an essential and integral part of us has revolutionized modern biology. The past decades have dramatically enriched our understanding of the holobiont concept, a mutual co-evolutionary relationship between the eukaryotic, prokaryotic and viral counterparts of an organism. The fundamentals of this concept of symbiotic co-existence rely on mutual requirements of the host and its microbiome and is regulated tightly by complex interactions. Of the distinct niches colonized by our microbiota within or on us, the gastro-intestinal tract harbours the most complex microbiota, consisting of bacteria, fungi, viruses, archaea and protozoa, and acts as a hotspot for host-microbe interactions (Kundu et al., 2017). The host through evolution and adaptation has developed diverse mechanisms to distinguish between the microbial symbionts and pathogens and respond accordingly by balancing between tolerance and inflammation. The first step of this interaction is mediated by pattern recognition receptors (PRRs), which sense microorganisms through conserved molecular structures. Several families of PRRs have now been well studied, including the Toll-like receptors (TLRs), the nucleotide-binding oligomerization (NOD)-like receptors (NLRs), the C-type lectin receptors, and the RIG-I-like receptors (RLRs) (Thaiss et al., 2016). Once the microbial signatures are recognized by the host, usually a transcriptional response follows, which determines the outcome of this interaction and is critical for maintaining the balance between homeostasis and inflammation. It is at this stage that members of the nuclear factor kappa B (NF-κB), play a crucial balancing act by maintaining tolerance towards the endogenous symbionts, hence establishing homeostasis while activating inflammatory pathways in response to abnormal changes in the microbiome, “dysbiosis” or pathogenic invasion. Most of the cellular PRRs such as TLRs, NLRs and RLRs after sensing microbial signatures follow distinct pathways, which ultimately converge to stimulate NF-κB, suggesting NF-κB’s central role in host response to microbes (Kawai and Akira, 2009). The balance in NF-κB response to microbial signatures becomes crucial for host health particularly during ageing and is often linked to ageing associated diseases. Ageing is associated with an overall decline in host organ functions, which changes host requirements and the dynamics between the host and its microbiota, leading to alterations in microbiome composition and diversity. These age-related microbiota transmutations can either be beneficial with the enrichment of health associated microbes and promote healthy ageing or may lead to severe imbalances leading to a potentially detrimental condition, termed dysbiosis (Kundu et al., 2017; Kundu et al., 2019). Ageing-associated dysbiosis usually triggers host immune responses, particularly the innate arm of it, since adaptive immunity typically declines with age, a phenomenon termed immunosenescence. This elevated basal level of innate immune responses during ageing leads to sustained inflammation, a condition known as inflammaging, which contributes to increased risk of developing age-related diseases. NF-κB signaling is a central player in this process as it integrates microbial cues via PRRs and in turn orchestrates innate immune responses. Thus, elevated constitutive activation of NF-κB during ageing, a fallout of microbial dysbiosis is associated with several age-related pathologies including chronic inflammatory diseases and many types of cancers.

## Changes in Gut Microbiome Dynamics With Host Age

Our gut microbiota is composed of a complex array of microorganisms, especially the commensals and mutualists, which exist in a symbiotic relationship with the host and have co-evolved to serve multiple roles and responsibilities (Kundu et al., 2017; Ragonnaud and Biragyn, 2021). They play a vital role in the maintenance of intestinal integrity, regulation of host immunity and act as a protective barrier against pathogens. Research using germ-free mice has greatly enriched our knowledge on the functional importance of gut microbiota, which aid in host digestion, absorption, and assimilation of food, generation of essential vitamins and minerals, and metabolism of undigested fibers into short chain fatty acids (SCFAs) (Carmody and Turnbaugh, 2012; Sommer and Bäckhed, 2013). We have recently shown that our gut microbiota regulates host blood brain barrier, immune responses, muscle functions, hippocampal neurogenesis, and intestinal growth and development (Braniste et al., 2014; Huang et al., 2019; Kundu et al., 2019; Lahiri et al., 2019). These commensals are the sole producers of SCFAs that can inhibit the colonization of other pathogenic bacteria and promote the production of protective mucus (Gill et al., 2006; Honda and Littman, 2016). These microbe-derived SCFAs are also an excellent energy source for epithelial and immune cells and can cross the gut epithelium to promote immune tolerance by inducing the anti-inflammatory FoxP3+ regulatory T cells (Tregs) and stimulating the production of TGF-β. Gut microbiota therefore play a crucial role in maintaining homeostasis and regulating the normal physiological processes of the host (Atarashi et al., 2013).

The composition of gut microbiota varies amongst individuals or within the same individual across age, playing distinct functional roles. It has the capacity to integrate external and intrinsic signals such as diet, geography, season, health status and age of the host and respond accordingly by changing its composition and community structure (Bosco and Noti, 2021; O'Toole and Jeffery, 2015). In addition, the microbiome being flexible, can be manipulated by dietary interventions and medications such as antibiotics. A healthy adult gut contains a typical population of Bacteroidetes and Firmicutes in dominance, while phyla such as Proteobacteria and Actinobacteria are maintained in relatively low proportions (Xu et al., 2019). However, a significant difference between the gut microbial composition of young adults and the elderly is noticed possibly because the microbiota continually changes to adapt with the ageing host, undergoing a progressive decline in body functions. Research from our lab showing dramatic changes in gut microbiota between inbred young and old mice kept under identical dietary and environmental conditions suggests that age-related host intrinsic factors play a key role in these gut microbiota changes (Kundu et al., 2019). In the elderly and certain age-associated diseases, a shift in the microbial ratio has been observed. There is a significant reduction of beneficial commensals such as Bacteroidetes, Firmicutes and Bifidobacterium and a parallel increase in harmful pro-inflammatory pathobionts belonging to Proteobacteria and Enterobacter. These undesirable changes in our gut microbiota are typically described as “dysbiosis” which impairs the integrity of the gut epithelium and increases intestinal permeability (O'Toole and Jeffery, 2015; Arumugam et al., 2011; Magrone and Jirillo, 2013; van Tongeren et al., 2005). Alterations and imbalance in the composition and diversity of the gut microbiota can trigger host immune responses and precipitate sustained inflammation due to dysregulation of homeostasis between the gut epithelium, gut-associated lymphoid tissue (GALT), and gut microbiota. Gut dysbiosis in the elderly leads to increased frailty and risk of age-related diseases (Kundu et al., 2017).

Several organism models have been used to understand the association between gut dysbiosis and ageing. In *Drosophila melanogaster* and African Turquoise Killifish, ageing was found to be associated with an increased abundance of Proteobacteria and reduction in Firmicutes, leading to an increase in the levels of inflammatory markers and loss of intestinal integrity (Clark et al., 2015; Smith et al., 2017). Further recolonization of middle-aged, antibiotics treated Killifish with the gut microbiota from young fish led to a significant increase in lifespan and a delay in age-dependent loss of activity (Clark et al., 2015; Smith et al., 2017). In germ-free mice, age-related inflammation and intestinal dysfunction were found to be significantly less as compared to conventionally raised mice bearing a typical gut microbiota. Recolonization of germ-free mice increased the intestinal permeability and inflammatory markers (Thevaranjan et al., 2017). All these observations suggest that changes in the gut microbial composition is a hallmark of ageing.

In *C. elegans*, it was observed that inhibition of folic acid synthesis in *E. coli* with the sulfonamide, sulfamethoxazole, prolongs the lifespan of the nematode without influencing bacterial growth. Another sulfonamide, sulfadiazine, has been shown to extend the lifespan of rodents and addition of the bacterial folate precursor, p-aminobenzoic acid (PABA), reversed the effect, implicating bacterial folate synthesis as a regulator of longevity (Virk et al., 2016). Furthermore, the sulfonamide drug, sulfasalazine is of clinical benefit in patients with Crohn’s disease and ulcerative colitis by reducing inflammation. This observation is important considering the association of these disorders with dysbiosis and enrichment of Proteobacteria (Peppercorn, 1990). Summarizing these points, it can be suggested that bacterial folic acid synthesis can be a potential driver of ageing. However, it still remains a question whether inhibition of bacterial folate synthesis could be clinically used as a target to delay ageing, which warrants further studies (Bannister et al., 2014). Orally administered drugs also have the capability to interact with gut microbiota. The oral antidiabetic drug metformin is proposed to slow ageing in humans. It causes a strain-specific increase in the lifespan of *C. elegans*, mainly by altering the metabolism of bacteria and bacterial gene expression. More research is however required to determine the association between metformin and ageing (Romdhoniyyah et al., 2021).

Surprisingly, a recent study demonstrates an enrichment of beneficial bacterial families such as Lachnospiraceae, Ruminococcaceae, Bifidobacterium, Akkermansia and Bacteridaceae in the gut microflora of healthy centenarians. Whereas harmful pathobionts like Proteobacteria were a minority. These microbial changes had probably helped them escape from major age-related diseases and achieve an overall good health (Biagi et al., 2016). Thus, targeting age-related gut dysbiosis could emerge as an effective therapeutic strategy to maintain a balanced immune response during ageing and protect against several age-related diseases.

## The Nuclear Factor Kappa B Family and Its Response

NF-κB, a family of transcriptional factors, regulates a large number of genes involved in multiple cellular processes, including innate immune and adaptive immune responses, cellular proliferation, differentiation, and genome stability (Liu et al., 2017). The connection between NF-κB and microorganisms dates back to its discovery by Ranjan Sen and David Baltimore in 1986, where they elegantly exhibited bacterial lipopolysaccharides (LPS) as a major inducer of NF-κB (Sen and Baltimore, 1986). Ever since, this pleiotropic transcription factor complex, its interactions, and functions have been extensively studied (Sen and Baltimore, 1986; Baeuerle, 1991; Baeuerle and Henkel, 1994; Siebenlist et al., 1994; Baldwin, 1996; Hayden and Ghosh, 2004; Karin and Greten, 2005; Perkins, 2007). There are five NF-κB family members in mammalian cells including RelA (p65), RelB, c-Rel, p50/p105 (NF-κB1) and p52/p100 (NF-κB2), which can form different NF-κB complexes as homo- or heterodimers. The NF-κB complexes are retained in the cytoplasm by the binding of inhibitory IκB proteins, typically by IκBα, IκBβ, and IκBε, the “classical” cytoplasmic inhibitors of NF-κB. These three inhibitors function by masking a conserved nuclear localization sequence, found in the Rel homology domain of the NF-κB subunits. Additionally, p105/IκBγ and p100/IκBδ, the precursors to mature NF-κB subunits p50 and p52, respectively, have also been clearly shown to function as cytoplasmic NF-κB inhibitor proteins (Savinova et al., 2009). While, Bcl-3 and IκB ζ appear to function primarily in the nucleus as modulators of specific NF-κB-dependent gene expressions and are consequently referred to as “nuclear IκB” proteins (Perkins, 2007). Phosphorylation of these IκB proteins, which may occur via a diverse array of stimuli, leads to their ubiquitination and breakdown in the proteasomes. This crucial phosphorylation step of IκBs is driven by kinases, the IKKs (IKKa and IKΚb) and NIK (NF-κB-inducing kinase) (Perkins, 2007). An essential NF-κB regulatory subunit of the IKK complex, NEMO governs the activation of IKK kinase complex (Sebban et al., 2006). After the phosphorylation of IκBs by IKKs, the NF-κB complex translocates into the nucleus where it activates the transcription of a number of genes, especially inflammatory genes (Perkins, 2007).

NF-κB activation involves two major signaling pathways, the canonical and noncanonical pathways, which although mechanistically different, both regulate immune and inflammatory responses, particularly those directed towards microbes. Considering the vast repertoire of microorganisms that constitute our microbiome, it is captivating to understand how the distinct components of the microbiota regulate NF-κB. Apparently, NF-κB activation by microbes is tightly regulated and this determines two possible outcomes, if homeostasis is maintained or if inflammatory pathways are activated leading to disease. For instance, NF-κB activation has been shown to regulate intestinal epithelial homeostasis via regulation of β-defensin 114 (Su et al., 2021). Components of the gut microbiota and their effector proteins can either activate or suppress NF-κB signaling pathway, thus maintaining the host-microbiota symbiosis and barrier integrity, especially in the intestinal milieu (Wells et al., 2010). For example, Firmicutes such as *Lactobacillus reuteri* can inhibit NF-κB response by preventing the degradation of its inhibitors and suppress TNF stimulation (Iyer et al., 2008). Probiotics and commensal microbes, especially Firmicutes have been shown to modulate NF-κB signaling at multiple levels including inhibition of processes such as TLR induction, transcriptional activation, phosphorylation, ubiquitination and proteasomal degradation of its inhibitor-IκBα, nuclear translocation and DNA binding of the p50/p65 isoforms, thus influencing homeostasis (Bhardwaj et al., 2020). Although speculative, the microbiome shift from Firmicutes that dominate the gut microbiome of young adults toward Bacteroidetes together with enrichment of Proteobacteria in the elderly may significantly impact NF-κB signaling and in turn disrupt homeostasis during ageing (Claesson et al., 2011). In contrast, pathobionts or pathogens are often equipped with secretion systems, which facilitate the release of virulence factors and effector proteins that can modulate immune responses associated with NF-κB signaling (Rahman and McFadden, 2011). The pathobionts or opportunistic pathogens employ diverse mechanisms to manipulate host NF-κB response that are distinct from the commensals (Rahman and McFadden, 2011). For instance, enterohemorrhagic *E. coli* can either activate or suppress NF-κB through T3SS-dependent or independent mechanisms, possibly through TLR-activation (Nobe et al., 2009), while *Pseudomonas aeruginosa* inhibits NF-κB signaling pathway by secreting a small molecule called N-(3-oxo-dodecanoyl) homoserine lactone (Kravchenko et al., 2008). Dendritic cells (DCs) from gut-associated lymphoid tissue are key regulators that finetune NF-κB response to symbionts and pathogens. Metabolites such as SCFA released by symbionts can induce tolerance by suppressing NF-κB signaling and promoting tolerogenic DCs and IL-10-producing TREGs. In contrast, DCs stimulate a pro-inflammatory response upon pathogen encounter by hyper-activating NF-κB signaling and promoting mesenteric Th1/Th17 cell differentiation, which leads to the release of pro-inflammatory cytokines- IL-6, TNF-α, IFN-γ, IL-17, neutrophil infiltration and stimulation of B lymphocytes to express IgG (Iacob et al., 2019). Thus, uncontrolled activation of the NF-κB pathway leads to activation of innate and adaptive immune responses, which may increase the extent and severity of inflammation and disease (Yan and Polk, 2010). Such unrestrained, chronic inflammation often leads to severe tissue damage, autoimmune diseases and cancer initiation, due to excessive cellular stress and accumulation of DNA damage.

## Microbe-Associated Molecular Patterns That Respond to Microbial Cues and Influence Nuclear Factor Kappa B Signaling

One of the major flanks of host immune response against microbes and pathogens is innate immunity, which induces the expression of effectors such as pro-inflammatory cytokines and interferons. However, less was known about its precise molecular mechanisms until recently. The discovery of the first PRRs, the toll-like receptors (TLRs) has provided a clearer picture about the process and gave a better understanding of the fundamentals of innate immunity (O'neill et al., 2013). TLRs are prototype PRRs that recognize microbe-associated molecular patterns (MAMPs), such as lipopolysaccharide (LPS) and lipoteichoic acid (LTA) or danger-associated molecular patterns (DAMPs) from damaged tissues and orchestrates host defense against infections by establishing a link between innate and adaptive immunity (Takeda et al., 2003). TLRs contain a leucine rich ectodomain mediating the recognition of PAMPs, a transmembrane domain, and cytosolic Toll-IL-1 receptor (TIR) domains activating the downstream signaling pathways. Till date, 10 and 12 functional TLRs have been identified in humans and mice respectively. Each TLR detects distinct PAMPs derived from viruses, bacteria, fungi, and parasites. These include lipoproteins (recognized by TLR1, TLR2, and TLR6), double-stranded RNA (TLR3), lipopolysaccharide (TLR4), flagellin (TLR5), single-stranded (ss) RNA (TLR7 and TLR8), and DNA (TLR9) (Akira et al., 2006). This is partly due to their spatial distribution for instance, TLR1, TLR2, TLR4, TLR5, and TLR6 are localized on the cell surface whereas TLR3, TLR7, TLR8, and TLR9 are expressed within intracellular vesicles (Blasius and Beutler, 2010). The microbial ligands recognized by the TLRs are possessed by most microorganisms and not exclusive to pathogens, therefore TLRs maintain a fine balance by regulating immune tolerance against beneficial commensal microbes (Larsson et al., 2021). This phenomenon is in part regulated by a process termed endotoxin tolerance (ET), which potentiates hypo-responsiveness to LPS stimuli upon subsequent exposures to low levels of LPS (Bauer et al., 2018). Moreover, the physical proximity of microbes to cells expressing TLRs is another factor that regulates the balance between tolerance and inflammation. Under steady state conditions, the commensals are kept at bay by various factors such as the mucous layer, antimicrobials peptides, whereas pathogens by virtue of their virulence factors escape these barriers leading to uncontrolled inflammation through TLR activation.

Once TLRs recognize the respective PAMPs, they recruit a set of TIR domain-containing adaptor proteins, such as myeloid differentiation primary response-88 (MyD88), TIRAP, TRIF, or TRAM and initiate downstream signaling pathways that finally lead to inflammatory responses through recruitment of immune cells and release of cytokines (Kawai and Akira, 2010). A key player in the TLR signaling is the adaptor protein, MyD88, which initiates a cascade of signaling events finally leading to the activation of NF-κB, interferon regulatory factor (IRF) and activator protein-1 (AP-1). Lack of MyD88 has been shown to significantly impact the composition of intestinal microflora, displaying the association between TLR signaling and microbial community structure (Kawai and Akira, 2010). Interestingly, several recent reports indicate that human TLR functions decline in specific immune cells with age (Della Bella et al., 2007; van Duin et al., 2007; Shaw et al., 2011), which could account for the changes in the microbial composition observed in aged individuals compared to young.

Another major arm of PRRs that play an important role in host response to microbiota is the oligomerization domain containing NOD-like receptors (NLRs). There are 22 different NLRs in humans and amongst them NOD1 (NLRC1) and NOD2 (NLRC2), the founding members of the NLRs, sense bacterial peptidoglycans and play a pivotal role in tissue homeostasis and host defense against bacterial pathogens (Philpott et al., 2014). Notably, NOD1 and NOD2 have been detected in the plasma membrane at the infection sites (Kufer et al., 2008) and upon activation by peptidoglycans both NOD1 and NOD2 induce the phosphorylation of their common adaptor receptor-interacting protein kinase 2 (RIP2). (Inohara et al., 1999; Ogura et al., 2001; Chin et al., 2002). Phosphorylation of RIP2 through a complex process ubiquitinates NEMO which instigates the activation of the canonical NF-κB pathway by phosphorylating IKKα and IKKβ and inducing the proteasomal degradation of IκBα (Saxena and Yeretssian, 2014). NOD2 recognizes cytosolic muramyl dipeptide (MDP) found in the peptidoglycan of all bacteria and has been recently been shown to protect intestinal stem cells from ROS mediated cytotoxic stress, which is critical in maintaining intestinal homeostasis (Levy et al., 2020).

## Importance of Nuclear Factor Kappa B Signaling in Homeostasis

NF-κB is activated by a wide range of stimuli and is a key regulator of cellular responses in both physiological and pathological conditions. Almost all the receptors of innate and adaptive immune systems activate NF-κB transcription factors to mediate host responses (Liu et al., 2017). On one hand it binds to the target sequences of various promoters and induces the expression of a variety of pro-inflammatory genes responsible for immune reactions including the ones encoding cytokines, chemokines, adhesion molecules, enzymes and antimicrobial peptides, and also the genes that drive cell differentiation and proliferation. On the other hand, it protects cells from apoptosis by inducing the expression of anti-apoptotic genes including BCL-XL and FLICE-like inhibitory protein (FLIP) and members of the inhibitors of apoptosis (IAP) family (Micheau and Tschopp, 2003). NF-κB is also shown to inhibit the accumulation of reactive oxygen species (ROS) by upregulation of proteins with antioxidant functions such as manganese superoxide dismutase (MnSOD) and ferritin heavy chain, thereby protecting the cells from necrosis (Warner et al., 1996; Pham et al., 2004). In addition, it is responsible for the activation of inflammasomes, which are multiprotein complexes expressed in various cell lineages and performs diverse functions for maintaining homeostasis, such as release of soluble pro-inflammatory molecules, effector cell activation and death of the affected cell through caspase-1 activation, phagosome maturation and autophagy (Wlodarska et al., 2014; Broz and Dixit, 2016). Deletion of IκB kinase b (IKΚb) in dendritic cells of mice has been shown to dysregulate immune homeostasis and development of spontaneous autoimmunity, suggesting the involvement of NF-κB in immune homeostasis and tolerance (Baratin et al., 2015). Another important mechanism of NF-κB regulation is through microRNAs, which target the 3′ untranslated region of mRNA and thereby decrease protein expression. Several microRNAs have been shown to be induced by NF-κB-dependent transcription. One of them being miR-146, which targets the expression of IRAK-1 and TRAF6 and downregulates the MyD88-dependent NF-κB response (Wu et al., 2018).

The canonical pathway of NF-κB signaling is activated by tumour necrosis factor, IL-1, and TLR ligands such as LPS. It plays an important role in the expression of genes involved in inflammation, cell proliferation, invasiveness, survival, angiogenesis, and metastasis (Shostak and Chariot, 2015; Piotrowski et al., 2020). Whereas, the noncanonical pathway is activated by lymphotoxin (LT), receptor activator of NF-κB ligand (RANKL), CD40 ligand (CD40L), and B cell activating factor of the TNF family (BAFF) and plays an essential role in the expression of genes that regulate the development of secondary lymphoid organs and related structures in the tissue microenvironment (Baratin et al., 2015).

Under normal physiological conditions, NF-κB plays a dual role. It directs the immune cells to elicit a response against any foreign element. At the same time, it protects the non-immune cells like the epithelial and stromal cells from the innate immune responses that are activated. Thereby, it ensures that tissue integrity and homeostasis are maintained and restores the ability of the immune system to respond to potential stressors (Pasparakis, 2009). For instance, our hepatocytes are being regularly exposed to several toxic substances including xenobiotics and microbial endotoxins that reach the liver through the portal circulation. These substances can damage the parenchymal cells and trigger the Kupffer cells to produce pro-inflammatory cytokines. NF-κB activation provides signals that protect the hepatocytes from potential degeneration. Inhibition of NF-κB sensitizes hepatocytes to death-inducing signals that result in inflammation and compensatory hepatocyte proliferation, ultimately resulting in the development of hepatocellular carcinoma. Thus, NF-κB signaling is critical in maintaining hepatic homeostasis (Luedde and Schwabe, 2011).

The intestinal epithelium being directly exposed to the gut microbiota acts as a protective barrier by producing mucus and antimicrobial peptides and plays a vital role in maintaining immune homeostasis (Coleman and Haller, 2018). They express several PRRs, which after recognizing microbial cues trigger the activation of NF-κB and other proinflammatory pathways. NF-κB inhibition results in increased damage of the epithelial cells and decreased expression of antimicrobial peptides, thereby destroying the integrity of the epithelial barrier and paving the way for the commensal bacteria to invade the mucosa (Pasparakis, 2008). Therefore, commensal bacteria are believed to regulate NF-κB activity at the intestinal epithelial interface and thereby affect the mucosal immune balance. Moreover, several cytokines are known to influence intestinal epithelial NF-κB activity, thereby making tight regulation of NF-κB signaling critical under both steady-state conditions as well as during activation of mucosal immune responses. Reasonably, severe imbalances in NF-κB activity has been implicated in diseases such as inflammatory bowel disease (IBD), Crohn’s disease as well as gastrointestinal cancers (Ellis et al., 1998; Schreiber et al., 1998; Coleman and Haller, 2018).

Another organ in direct contact with a complex microbiota is our skin, where homeostasis is maintained by the cross-talk between epidermal keratinocytes and dermal immune cells. NF-κB inhibition in mouse epidermis disturbs skin homeostasis and triggers TNF-dependent skin inflammation, epidermal hyperplasia, and the development of squamous cell carcinoma. Moreover, NF-κB signaling blockade perturbs the communication between the epidermis and the dermis and initiates an inflammatory response that resembles a wound-healing reaction (Coleman and Haller, 2018).

**FIGURE 1:**
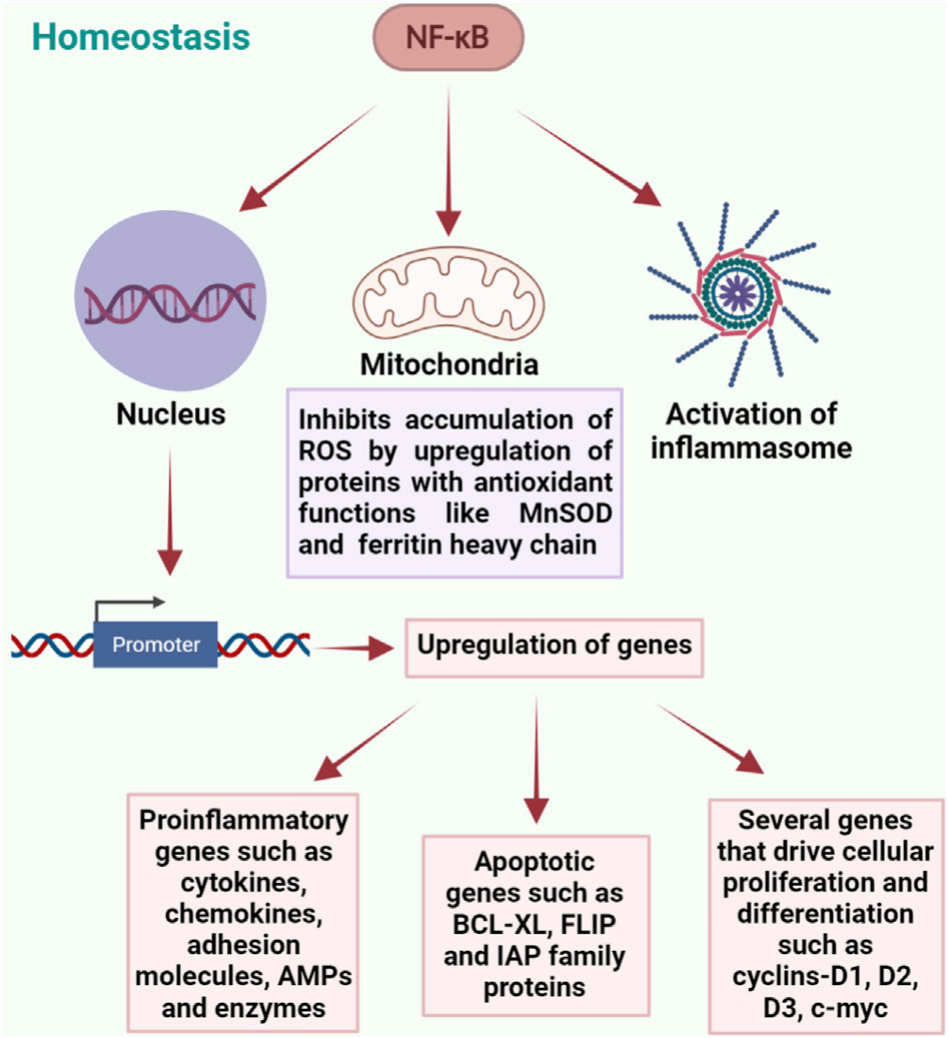
Diverse roles of NF-κB in regulation of homeostasis. NF-κB plays a central role in maintaining homeostasis through a complex network of interactions involving regulation of gene expression, maintaining mitochondrial ROS levels and activation of inflammasomes.

It is therefore evident that NF-κB is highly beneficial for the survival of cells and also for conferring protection against infection and trauma. However, prolonged or uncontrolled activation of NF-κB signaling could be detrimental and requires timely termination. Even though NF-κB activity is tightly regulated by feedback mechanisms, constitutively elevated levels of NF-κB are associated with several chronic inflammatory diseases and many types of cancers. Hence for persistent homeostasis, it is crucial to maintain this delicate balance in order to remain tolerant towards our endogenous symbiotic microbiota, while retaining the capacity to elicit appropriate immune responses towards pathogens (Lawrence, 2009; Coleman and Haller, 2018; Taniguchi and Karin, 2018). Together, these findings demonstrate the critical role of NF-κB in maintaining homeostasis (Figure 1). However, detailed molecular mechanisms by which NF-κB regulates normal tissue homeostasis require further investigation.

## Ageing Associated Changes in Microbiota and Nuclear Factor Kappa B

Ageing is a complex physiological process involving a progressive decline in body functions. Ageing is associated with cellular senescence which often leads to reduced capacity in maintaining homeostatic processes and stress responses leading to increased risk of diseases (Hoare et al., 2010). With the advancement of modern medicine, today, longevity has increased considerably leading to a steep increase in the global ageing population (Castro-Mejía et al., 2020). In the early 1900s, Russian microbiologist, Elie Metchnikoff had proposed that ageing and frailty are mainly caused by the destruction of human tissues due to the accumulation of putrefying bacteria and their toxins and suggested that a simple diet of fermented milk along with a healthy lifestyle can neutralize these toxins and slow down the ageing process. His idea eventually helped in realizing the importance of microorganisms in the process of ageing and age-related pathologies (Metchnikoff, 2004). Indeed, our group and others have shown that gut microbiome evolves with age in humans and experimental animals and this is possibly due to age-associated changes in diet, excessive use of medications, lack of physical activity, living environment as well as host intrinsic signals (Kundu et al., 2017; Kundu et al., 2019). For instance, Claesson et al. (2011) showed Bacteroides, Alistipes, and Parabacteroides occupied more than half of the ageing core microbiomes, whereas they contribute to just 8%–27% in young. During ageing, the efficiency of immune response declines, leading to an uncontrolled microbial growth, reduction in microbial diversity and enrichment of pathobionts. It is at this stage that the homeostatic balance between the major microbial counterparts in the gut microbiome, the mutualists, commensals and pathobionts collapses leading to dysbiosis. The enrichment of pathobionts is associated with a consequent influx of bacterial enzymes, toxins as well as reactive oxygen/nitrogen species from infiltrating inflammatory cells that cumulatively degrades the tight junction proteins and initiate epithelial cell death and denudation. In parallel, age-related altered intestinal architecture and turnover of epithelial cells, functional changes in Paneth cells (Nalapareddy et al., 2017), reduced expression of epithelial tight junction proteins (Ren et al., 2014) together result in increased permeability and impairment of intestinal integrity leading to the translocation of pathobionts and their endotoxins across the intestinal epithelium into the circulation (Britton and McLaughlin, 2013; Clark et al., 2015). This dysbiosis-associated increased microbial translocation across the gut epithelium, eventually leads to massive TLR activation, in enterocytes and immune cells, particularly macrophages, which essentially engage NF-κB dependent antimicrobial effectors such as inflammatory cytokines and chemokines including TNF-α, IL-6, IL-8, and IL-23 and promotes persistent intestinal inflammation (Biagi et al., 2013; Thevaranjan et al., 2017). Moreover, the increased basal levels of circulating TNF-α during ageing directly influences NF-κB signaling, considering TNF receptors can activate NF-κB complex following pro-inflammatory signals (Rahman and McFadden, 2011; Thevaranjan et al., 2017). This corroborates with an ageing-associated increase in microbial lipopolysaccharide levels which is a major inducer of NF-κB activation (Kim et al., 2016). Moreover, toxins from pathogenic microbes such as *Bacteroides fragilis* have been shown to accumulate during ageing in humans, which can stimulate IL-8 secretion via NF-κB activation (Wu et al., 2004). Simultaneously, the population of specific beneficial microbial communities such as members of *Verrucomicrobia phylum*, which maintain intestinal barrier integrity and thereby prevent inflammation, decline in the elderly, leading to intestinal barrier breach and inflammation (Ragonnaud and Biragyn, 2021). Together, these events associated with changes in the microbiota as well as increased levels of oxidative stress during ageing lead to a chronic low-grade inflammation, a condition termed as “inflammaging” (Franceschi et al., 2000), which is a common contributor to ageing related diseases. When it comes to controlled inflammation, the inflammatory responses are tightly regulated and ideally end immediately after the clearance of the infectious agent or healing of damaged tissues. However, during inflammaging, the balance between inflammatory and anti-inflammatory responses is typically disrupted leading to non-resolving inflammation (Nathan and Ding, 2010). The hallmark of inflammaging is abnormal activation of innate immunity and elevated pro-inflammatory status, which could be regarded as the common biological factor responsible for the age-related diseases including type 2 diabetes, cardiovascular diseases, cancer, atherosclerosis, Alzheimer’s, and Parkinson’s disease (Cevenini et al., 2013).

Although inflammaging is a complex process, which involves an array of molecular mediators and interactions, the major defense signaling mechanisms involving innate immunity are evolutionarily conserved, where NF-κB signaling acts as a central regulator. It has been shown that the DNA-binding pattern of NF-κB complexes does not change between young and old rodents (Salminen et al., 2008). Moreover, ageing does not influence the protein or mRNA levels of key IκB inhibitors, IKK subunits, or IκB components (Salminen et al., 2008). However, the nuclear localization of p52 and p65, NF-κB components are significantly increased in ageing rodents (Salminen et al., 2008). These findings demonstrate that there’s an increase in NF-κB components entering into the nuclei during inflammaging, which activates downstream pro-inflammatory cytokines, such as IL-6, TNF-α and IL-1α, and reshapes the immune system during ageing. Thus, targeting NF-κB signaling using microbiota-based strategies to combat inflammaging is an attractive futuristic clinical option to promote healthy ageing. Interventions such as fecal microbiota transplant (FMT) could be a prospective option, considering the recent findings by our group and others using animal models, pointing out that microbiome transplant can potentially influence the ageing process (Smith et al., 2017; Kundu et al., 2019). Another attractive modality aimed at targeting NF-κB signaling is functional food products containing pre- and probiotics known to modulate inflammation via NF-κB. A recent review by Vincenzi et al. (2021) nicely outlines several probiotics and bacterial strains influencing NF-κB functions, which ultimately protect from inflammation.

## Crosstalk Between Microbiota and Nuclear Factor Kappa B Signaling in Ageing Associated Diseases

**FIGURE 2:**
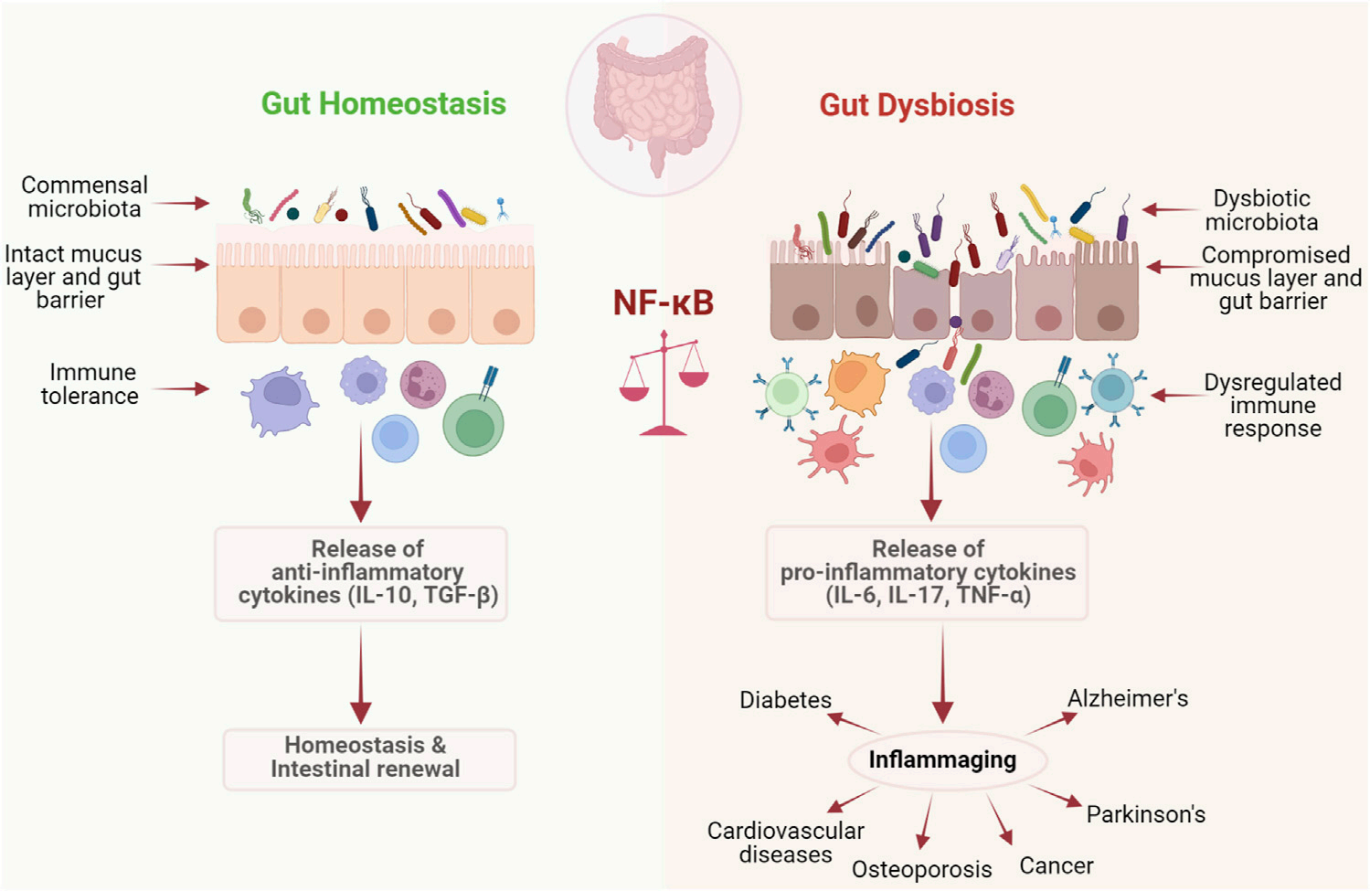
NF-κB maintains the fine balance between homeostasis and dysbiosis. NF-κB plays a dual role if host response to microbiota. It plays a critical role in maintaining homeostasis by promoting immune tolerance against commensal gut microbiota, while under dysbiotic conditions it drives inflammation, leading to a number of chronic diseases.

With the advancement of sequencing tools, especially metagenomics, we are now able to establish the link between diseases that stem from ageing and abnormal changes in the gut microbiome. Modern computational techniques and the development of prediction models further connect these age-related diseases with inflammaging and signaling pathways associated with it, particularly NF-κB signaling. Several studies have shown that accumulation of cellular damage can trigger the process of ageing by promoting apoptosis and cellular senescence. Stress mediated activation of NF-κB signaling usually occurs through the canonical pathway rather than the non-canonical pathway during ageing (Tilstra et al., 2011). In this process, several other pathways like insulin/IGF-1, FOXO, SIRT, p53, and mTOR crosstalk with NF-κB signaling, making it a highly complex process (Niedernhofer and Robbins, 2008). Studies involving the mapping of combinatorial cis regulatory motifs that are responsible for age-dependent gene expression across different tissues and organisms have suggested that the transcription factor NF-κB is predominantly associated with ageing (Adler et al., 2007). It has been observed that inhibition of NF-κB for 2 weeks in the epidermis of chronologically aged mice have reverted the gene expression and tissue characteristics to that of young mice (Adler et al., 2007). Furthermore, overexpression of c-Rel and Rel-A/p65, the two subunits of NF-κB in cultured cells was shown to elicit a specific senescence-associated secretory phenotype, associated with overexpression of cytokines and chemokines like IL-6, IL-8, IL-7, MCP-2, MIP-3, ICAM, IL-1α, and IL1-β (Coppé et al., 2010). It has also been observed that, binding of NF-κB/p65 with DNA had increased by several fold with chronological ageing in several tissues such as skin, kidney, liver, brain, heart, blood vessels, and muscles (Helenius et al., 1996). Moreover, the effect of caloric restriction, which extends lifespan and protects against age-related pathologies, is primarily due to the inhibition of NF-κB signaling at the level of IKK through a reduction in oxidative stress and reactive oxygen species generation (Kim et al., 2002). Inflammation resulting from a combination of gut dysbiosis and NF-κB signaling activation is usually observed in several age-related pathologies such as cardiovascular, metabolic, and neurodegenerative disorders as well as cancer (Tilstra et al., 2011). This again portrays the contrasting roles of NF-κB in homeostasis and dysbiosis associated with ageing (Figure 2).

### Neurodegenerative Diseases

Ageing is typically associated with an increase in the incidence of neurodegenerative diseases like Alzheimer’s disease (AD) and Parkinson’s disease (PD). AD involves increased intracranial activity of inflammatory cytokines like IL-1β, IL-6, and TNF-α due to amyloid-β (Aβ) stimulation of NF-κB activity in the microglia (Combs et al., 2001). Interestingly, a direct microbial involvement in the development of a beta amyloid pathology has recently been shown by Harach et al. (2017). Another report indicates that the gut microbiomes of AD patients are compositionally distinct and typically display decreased microbial diversity compared to healthy individuals (Vogt et al., 2017). Overexpression of the amyloid precursor protein (APP) gene is associated with increased susceptibility to AD. APP is cleaved by the beta-secretase BACE1 and forms amyloid plaques in the brain of AD patients. Both BACE1 and NF-κB are increased in the brain of AD patients and NF-κB is responsible for upregulating BACE1 and APP genes (Chen et al., 2012). This process can be reversed by medications such as the antibiotic minocycline, suggesting a microbiota dependent NF-κB activation in the development of AD (Cai et al., 2011). The pathognomonic role of Aβ is now also being questioned with the discovery that Aβ acts as an antimicrobial peptide (AMP) in cell lines, nematode, and rodent models (Moir et al., 2018). In rats, NF-κB expression increases in normal ageing leading to the production of neurodegenerative pro-inflammatory enzymes COX-2 and iNOS (Kim et al., 2010). Suppression of the same by the anti-inflammatory *Lactobacillus pentosus* var. plantarum C29 reverses the process (Jeong et al., 2015).

PD is another neurodegenerative condition with increased inflammation and cytokine signaling in both the brain and CSF. Inflammation derived oxidative stress and cytokine toxicity leads to the misfolding of the protein, α-synuclein (syn) and its aggregation in the brain of PD patients (Mogi et al., 1994). The role of gut microbiota in PD development has been elegantly shown by Sampson et al. (2016) recently. They showed that colonization of a Syn-overexpressing mice with microbiota from PD-affected patients enhances physical impairments compared to microbiota transplants from healthy human donors (Sampson et al., 2016). Furthermore, the gut microbiomes of PD patients typically display an enrichment of pathobionts with a simultaneous decrease in beneficial bacteria. Immune activation in the enteric nervous system has been shown to initiate α-syn pathology, which then spreads to the substantia nigra in the brain through the vagus nerve. Thus, gut dysbiosis and leakiness due to bacterial endotoxins activate TLR4-mediated inflammatory cascades, which eventually leads to neurodegeneration in PD (Sampson et al., 2016; Perez-Pardo et al., 2019). Consequently, a considerable increase in the activation of p65/RelA has been observed in dopaminergic neurons of PD patients (Hunot et al., 1997), while administration of NF-κB inhibitors have improved the pathological changes in a murine model of PD (Flood et al., 2011).

### Metabolic Diseases

The incidence of metabolic diseases like Diabetes, which is typically associated with dysbiosis, increase dramatically with age. Type-1 Diabetes Mellitus (T1DM), involves autoimmune destruction of β-cells, which is mediated by cytokines like IL-1. Upon increased exposure to IL-1, NF-κB is activated, which translocates into the nucleus to further activate several genes responsible for β-cell destruction and death by apoptosis. Gut dysbiosis related NF-κB activation is also one of the key features associated with T1DM, leading to deregulated immune responses that further cause loss of intestinal integrity and increased microbial passage into circulation (Cardozo et al., 2001). This activates T-cells, which ultimately results in the destruction of pancreatic β-cells (Cardozo et al., 2001). Inhibition of NF-κB in mice has been shown to protect β-cells from streptozotocin induced T1DM (Eldor et al., 2006) while, upregulation of NF-κB signaling in hepatocytes is associated with the etiology of Type-2 Diabetes Mellitus (T2DM) (Cai et al., 2005). One of the likely reasons could be T2DM-associated dysbiosis, which shows a marked reduction in populations of the genera Bifidobacterium, Bacteroides, Faecalibacterium, Akkermansia, and Roseburia, with a simultaneous increase in Ruminococcus, Fusobacterium, and Blautia during T2D (Gurung et al., 2020). Reasonably, oral administration of members of the microbiota such as *Akkermansia muciniphila* exerted antidiabetic effects (Tilg and Moschen, 2014). The involvement of NF-κB in the pathogenesis of T2DM was considered following the observation that high doses of salicylates correct the hypoglycemic state in T2DM and aspirin further inhibits NF-κB by preventing the degradation of IκB (Kopp and Ghosh, 1994). NF-κB is also responsible for the expression of GLUT-2 transporter which is crucial for the transport of glucose inside cells (Norlin et al., 2005). The function of peroxisome proliferator-activated receptor gamma (PPAR-γ), a receptor that maintains glucose homeostasis, is also antagonized by NF-κB activation in mesenchymal cells (Suzawa et al., 2003). Moreover, TNF-α, which is regulated by NF-κB, phosphorylates insulin receptor substrate-1 (IRS-1) leading to insulin resistance. Neutralization of the same increases insulin sensitivity (Yang et al., 2009). Furthermore, translocation of bacterial components like lipopolysaccharides into the circulation triggers pro-inflammatory cytokine release and development of a chronic low grade inflammatory state, one of the characteristic features of T2DM. Together, these facts clearly suggest that NF-κB and gut dysbiosis are important players in the pathogenesis of T1DM, T2DM and diabetes associated complications (Hotamisligil et al., 1995).

### Cardiovascular Diseases

Hyperactivity of NF-κB signaling is one of the main etiologies of several cardiovascular disorders. Increased NF-κB activity has been observed in unstable atherosclerotic plaques of the coronary artery (Monaco et al., 2004). In a bacterial LPS induced apoE−/− mouse model, genetic suppression of NF-κB signaling was associated with a significant reduction in the size of atherosclerotic lesions (Cuaz-Pérolin et al., 2008). TNF-α, both an inducer and target of NF-κB increased ROS formation, apoptosis and endothelial dysfunction in rat carotid arteries, while TNF-α inhibition resulted in vasculo-protective effects, suggesting an association between NF-κB activation and ageing associated atherosclerosis (Csiszar et al., 2007).

### Bone Diseases

Osteoporosis, one of the major age-related problems is associated with decreased bone density, increased fragility of bones, and accelerated osteoclast formation due to the hyper-activity of NF-κB driven inflammatory cytokines such as IL-6, TNF-α, and IL-1 (Soysa and Alles, 2009). It has been observed that in patients with co-morbid conditions involving NF-κB hyperactivity such as HIV infection, hyper-IgE syndrome, rheumatoid arthritis, multiple myeloma, and inflammatory bowel disease, there is an increased risk for the development of osteoporosis (Ginaldi et al., 2005). Thus, like several other age-associated diseases, NF-κB may act through numerous mechanisms to promote osteoporosis. Studies have shown that inhibition of NF-κB, improves bone density in accelerated ageing mice and improves intervertebral disc integrity indicating an association between NF-κB signaling and osteoporosis (Ding et al., 2008). An interesting study by Sjögren et al. (2012), demonstrated that gut microbiota directly regulates bone mass as germ-free mice exhibit increased bone mass due to reduced number of osteoclasts per bone surface compared with conventionally raised mice. This suggests that age related osteoporosis may involve an interplay between microbiota and NF-κB, which requires further investigation. Altogether, targeting NF-κB through microbial manipulation could therefore have a great potential for the treatment of several chronic age-related diseases. But the benefits should overweigh the potential risks of NF-κB blockade.

### Cancer

Ageing is associated with a dramatic increase in the incidence of cancer, which is also the second most common cause of death globally, according to statistical data from the World Health Organization (Fitzmaurice et al., 2019). Considering the rapid rise in the elderly population, it is predicted that the global burden of new cancer cases may rise to about 24 million by 2035 (Pilleron et al., 2021). The two most common forms of cancer that are associated with microbes or dysbiosis are gastric and colorectal cancer. The discovery by Marshall and Warren that *Helicobacter pylori* is the potential cause of gastric cancer, established the initial link between bacteria and oncogenesis. Subsequent reports by several groups, including ours, demonstrated that NF-κB signaling is one of the main pathways that potentiate the bacteria mediated gastric oncogenesis (Mori et al., 2003; Kundu et al., 2011). Prolonged activation of NF-κB pathway can lead to increased cellular stress, DNA damage and alterations of the genetic and epigenetic states of damaged tissues, leading to a pro-tumorigenic microenvironment (Lawrence, 2009). The oncogenic potential of NF-κB mainly depends on the deregulated expression of genes involved in apoptosis, cell proliferation, cell cycle control, and cell migration. Constitutive activation of NF-κB promotes cellular proliferation and cell cycle progression through cyclins-D1, D2, D3, E, c-myc, and c-mycb. It also induces the expression of adhesion molecules such as ICAM-1, selectin and MMPs (Mori et al., 2003). Furthermore, it prolongs the survival of cells by suppressing apoptosis due to increased expression of the anti-apoptotic BCl-2 family genes and downregulation of PTEN. Alterations in NF-κB is seen in many human cancers, while tumour cells with constitutive activation of NF-κB usually show increased resistance to chemotherapy (Taniguchi and Karin, 2018).

Ageing has also been shown to significantly increase the risk of developing CRC, the incidence of which dramatically increases after 50 years of age in humans (Simon, 2016). Accumulating data indicate that CRC initiation and development are associated with abnormal changes in gut microbiota. We have recently shown that germ-free APC Min/+ mice, predisposed to develop cancer, display significantly reduced tumor load compared to their conventionally raised counterparts suggesting a direct role of gut microbiota in intestinal tumorigenesis (Li et al., 2012). Several clinical studies have further validated the concept of gut dysbiosis associated colon cancer. Some studies indicated a reduction in the abundance of certain genera such as Clostridium and Roseburia (Chen et al., 2013). While, other studies have shown overrepresentation of *Streptococcus gallolyticus*, *Fusobacterium*, *Escherichia coli (E. coli)* pks+, *Peptostreptococcus* and enterotoxigenic (LT+) *B. fragilis* (ETBF) (Hensler, 2011; Marchesi et al., 2011; Kostic et al., 2012; Dalmasso et al., 2014; Viljoen et al., 2015; Long et al., 2019). An increasing pool of data suggests that microbiota or its components and metabolites can modulate NF-κB signaling leading to CRC development (Kostic et al., 2013; Pallett et al., 2014; Tsoi et al., 2017; Yang et al., 2017; Chung et al., 2018; Long et al., 2019; Chen et al., 2020). For instance, *B. fragilis* toxin is known to trigger colonic tumorigenesis through an IL-17-dependent NF-κB activation in colonic epithelial cells (Chung et al., 2018). The anaerobic bacterium *Peptostreptococcus anaerobius,* which is often enriched in the gut microbiota of CRC patients, was shown to activate NF-κB and promote CRC through its surface protein PCWBR2-integrin (Long et al., 2019). *Fusobacterium nucleatum*, a member of the oral microbiome, has been reported to deregulate NF-κB signaling, as it overpopulates the colonic microbial niche during CRC. APC Min/+ mice exposed to *F. nucleatum*, showed accelerated intestinal tumorigenesis through nuclear translocation of NF-κB subunit p65, compared to untreated controls (Kostic et al., 2013). Additionally, Yang et al. (2017) showed that the activation of NF-κB by *F. nucleatum* was mediated through a TLR4 and MyD88 driven signaling pathway. Together it is evident, that regulation of the NF-κB signaling pathway by gut microbes plays a central role in cancer development, making NF-κB a potential therapeutic target for ageing-associated diseases like cancer.

## Conclusion and Future Perspectives

It is well established that dysbiosis of gut microbiota is associated with ageing and age-related pathologies. Hence, a better understanding of the age-related dynamic changes in gut microbiota and its effect on various cellular signaling pathways is essential for the development of therapeutic strategies that could delay the process of ageing or promote healthy ageing. Considering the difficulties in pursuing mechanistic studies in human subjects, studies involving germ-free, transgenic mice mimicking human ageing and associated diseases, where the gut microbiome could be manipulated are highly encouraged. Such studies will significantly enrich our current understanding on microbial involvement in the host ageing process and help decode the underlying molecular mechanisms. Much work has been done over the recent years to understand and characterize the interactions between the innate immune system and microbial pathogens and the crosstalk between gut microbiota and NF-κB signaling pathway has been implicated as one of the major determining factors of ageing and age-related diseases. Targeting the NF-κB signaling by exploiting the flexibility of the gut microbiome could therefore have a great potential in developing safe and effective clinical options for the treatment of several age-related pathologies. However, further studies are required to assess the risk-benefit ratio of NF-κB signaling manipulation.

Selective inhibition of NF-κB signaling could be utilized to develop potential anti-ageing or anti-cancer agents. Proteasomal inhibitors are one such class of compounds that prevent the degradation of IκB and thereby block NF-κB pathway, as exemplified by the drug bortezomib, which is being effectively used in the treatment of multiple myeloma (Sung et al., 2008). Several polyphenolic compounds such as anthocyanins, ellagitannins, and ellagic acid derivatives including the microbial metabolite urolithin (Uro-A and Uro-B) target the IKK complex and prevent the translocation of NF-κB to the nucleus thereby suppressing the production of pro-inflammatory cytokines (González-Sarrías et al., 2010; Karunaweera et al., 2015).

There is much scope to develop potential futuristic NF-κB signaling “modulators” by manipulating the gut microbiota using specific microbes of potential biological importance, which could reshape the microbiome-NF-κB axis and emerge as an effective therapeutic strategy to delay the ageing process or protect against age-related pathologies. For example, *Bacteroides thetaiotaomicron*, known to prevent the activation of NF-κB by promoting the nuclear export of NF-κB subunit RelA through a PPARγ dependent pathway (Kelly et al., 2004) can be a probable option. Such candidate microbes could be utilized to develop personalized medicine, in which elderly patient- or disease-specific dysbiotic signatures could be identified and manipulated as a potential therapeutic strategy. Taken together, a detailed understanding of age-related gut dysbiosis and its crosstalk with NF-κB is critical to develop futuristic therapeutic strategies and support the 21st century medicine in its attempt to promote health during ageing.
